# Rural Nurses’ Views on Breastmilk Banking in Limpopo Province, South Africa: A Qualitative Study

**DOI:** 10.3390/nursrep12040074

**Published:** 2022-10-16

**Authors:** Mantji Annah Mahlatjie, Makoma Bopape, Tebogo Maria Mothiba

**Affiliations:** 1 Department of Human Nutrition and Dietetics, University of Limpopo, Sovenga 0727, South Africa; 2 Executive Dean of Health Sciences, Professor, University of Limpopo, Sovenga 0727, South Africa

**Keywords:** donation, donated breastmilk, nurses, breastmilk banks, views

## Abstract

The development of breastmilk banks is being established among the African population, including in Limpopo Province. However, the views of nurses directly handling the donated breastmilk in the province remain unknown. This study was aimed at exploring and describing the views of nurses towards breastmilk banking in the Mankweng area, Limpopo Province. A qualitative, descriptive, and explorative study was undertaken at a tertiary hospital and a rural feeder clinic in the Mankweng area. Purposive sampling was employed to obtain participants for the interviews. One-on-one, semi-structured interviews were conducted to explore the views of these nurses. Data were analysed using Tesch’s open coding method, with the information obtained being grouped into different themes and sub-themes. Almost all nurses were willing to donate their breastmilk to the bank. However, receiving donated breastmilk for their own children seemed to be a challenge due to the safety of the donated breastmilk, uncertainty about the screening process, and cultural issues. Although donation of breastmilk appeared to be well supported by almost all the nurses, the use of donated breastmilk seemed to be not fully acceptable. Increased awareness about breastmilk donation and banking should be prioritised in the province.

## 1. Introduction

The World Health Organization (WHO) encourages exclusive breastfeeding for the first 6 months, which should be started within the first hour after birth. Breastmilk is regarded as the first choice for infant feeding and an incomparable way of providing ideal food for the healthy growth and development of infants [1]. Mother’s own milk (MOM) is nutritionally complete to meet the infant’s nutrient requirements. It has been shown to provide health benefits for all new-born infants including ones at high risk or with very low birth weights [2]. In the absence of the infant’s mother due to maternal illness, death, medication, disability, insufficient milk production, allergies, or prematurity, donated breastmilk from the breastmilk bank can become the ideal option [3,4].

The use of pasteurised donor human milk (PDHM) has become the standard of care for infants when MOM is unobtainable [5]. The WHO recommends receiving donor milk as being lifesaving and the best alternative for infants who are unable to obtain milk from their mothers [6]. In South Africa (SA), many new-born infants whose MOM is unavailable, especially preterm, low-birth weight, severely malnourished, and orphan neonates—are provided with donated breast milk from the banks [7]. Donated breastmilk (DBM) is considered to reduce morbidity and mortality among vulnerable infants [8]. Although it is incomparable to MOM, it is preferred over infant formula because it lowers the risks of sepsis, Necrotising Enterocolitis, diarrhoea, and feeding tolerance and reduces the length of stay in critical care [9]. In addition to the benefits of breastmilk to a child’s health, it is also beneficial to the mother by decreasing the risk of breast and ovarian cancers, lactation amenorrhoea, and postpartum weight retention [10]. In a study on the effectiveness of donor milk among helpless preterm and low-birth weight infants involving a sequence of cases in Ithemba lethu, SA, Kwazulu Natal province, there were no signs of adverse reaction to DBM [11]. Even though some of the immune properties are destroyed during the pasteurisation process, DBM is superior compared to infant formula [12,13].

There has been a global increase in the number of human milk banks, aiming to improve neonatal care. In the year 2020, there were 756 human milk banks in 66 countries, and the number is escalating in low- and middle-income countries (LMICs). Brazil is one of the leading LMICs to have set a standard for incorporating human milk banking into their larger healthcare system [14]. There has been an increase in human milk banks in other parts of the world, including in South Africa [7,15]. In SA, there are 70 functional human milk banks that are guided by the Human Milk Banking Association of South Africa (HMBASA) and the South African Breastmilk Bank Reserve [16]. Currently, Limpopo province has two functional breastmilk banks operating in Capricorn and Sekhukhune districts, guided by the HMBASA. These banks were established to provide optimal nutrition and immunological protection to vulnerable infants, such as those who are premature, severely malnourished, orphaned, or facing the risk of illness or death [8]. Donated breastmilk is not precisely equal to mother’s own milk due to some micronutrients, anti-infective factors, and other nutritional and immunologic factors lost during pasteurisation [10]. It does, however, retain the ability to inhibit bacterial growth, although it is slightly reduced in comparison to MOM [17].

Several studies have surveyed people’s views on donated breastmilk, mostly among healthcare workers in developing and developed countries, and yielded different results. A study in Australia (a developed country) revealed that healthcare professionals believed that DBM was superior when compared to formula, and preferred it as the best feeding option for preterm infants. Additionally, in the same study, more nurses accepted DBM than doctors [18]. A study conducted in Zimbabwe (a developing country) reported that most of the healthcare workers had sufficient knowledge regarding breastmilk banks and that participants would accept the DBM to be fed to their own children. The participants were willing to donate and mostly would encourage the mothers to donate and use donated milk from the bank [19]. A study conducted in Indonesia indicated low acceptability of DBM among healthcare workers and most mothers due to the fear of transmission of diseases from the donated milk [20]. In SA (Limpopo, Northwest and Kwazulu Natal provinces), healthcare workers were concerned about the safety of donated milk, particularly regarding HIV transmission and transportation of donated milk from the donor to the bank, which might affect the quality of the milk [4,21,22,23]. These concerns were only reported amongst breastfeeding women receiving healthcare services at Mankweng hospital and clinic [4]. Mankweng serves as a tertiary referral hospital in the Limpopo province. The hospital has, among others, neonatal ICU, Kangaroo Mother Care, and high care units. Limpopo province established the first breastmilk bank in Mankweng hospital in the year 2016, followed by another one in Sekhukhune district operating from 2019. The latter was part of strategies to reduce high child mortality rates and to protect, support, and promote breastfeeding for all mothers, including those living with HIV as outlined in the Tshwane declaration [24]. However, there remains a paucity of data documented in Limpopo province involving concerns of healthcare professionals, particularly nurses, regarding breastmilk donation in the Mankweng area. Healthcare professionals, especially nurses, have a responsibility to provide information about breastfeeding and breastmilk expression and are responsible for encouraging mothers to donate to the breastmilk bank, thus increasing the uptake of donor milk practices [16,18,25,26]. Antenatal education may provide sufficient time for mothers to prepare for lactation prior to admission of the infant to NICU post delivery [27]. This could assist in increasing awareness in developing countries, incorporating donor breast milk as a major aspect of collective maternal and child wellbeing strategies and thus closing the knowledge gaps [8,28].

## 2. Materials and Methods

### 2.1. Materials

Data collection tools in this research included an interview guide, voice recorder, and note book. These materials were used for data collection during interviews of nurses as participants.

### 2.2. Methods

#### 2.2.1. Research Design

A qualitative research method was applied, which enabled the researchers to deeply understand the views of the nurses regarding breastmilk banking. A explorative and descriptive study design was employed. The nurses were given the opportunity to explore and describe their views regarding breastmilk banking.

#### 2.2.2. Study Setting

Data were collected at the selected rural facilities: Mankweng hospital and Mankweng clinic, Limpopo Province. Mankweng hospital was chosen as it was the only hospital with an established breastmilk bank at the time of data collection, while Mankweng clinic was selected due to being a feeder facility nearby the established breastmilk bank. The clinic is situated 1.5 km away from the hospital and both health facilities are 30 km away from Polokwane town, the capital city of Limpopo province.

#### 2.2.3. Population and Sampling

Nurses who were employed at the clinic and neonatal ward in a hospital during the time of data collection served as the population for this study. Participants who were on duty on the day of data collection were purposively sampled. Male nurses and nurses who were on duty and attending to clients at the time of data collection were excluded from the study. The sampling process was conducted by the researchers until data saturation was reached.

#### 2.2.4. Data Collection

Data were collected over a period of three months from December 2016 to February 2017 at Mankweng hospital and Mankweng clinic. The researchers approached the nurses face to face and built a rapport by explaining the aim and purpose of the study. Private offices were provided by both institutions for the interviews to take place. In-depth, one-on-one interviews using a semi-structured interview style were conducted during data collection. The data collection tool consisted of two sections: A (demographic information) and B (interview guide). The interview guide was tested with the first participant at the clinic, the data of which were excluded from the main study. The demographic information included, among others, age and marital status of the nurses, while section B consisted of the interview guide, which contained different questions for the researchers to refer to and to remain focused on during the interview. The interviews were started with one question asked to guide the discussions, followed by probing questions to achieve greater clarity pertaining to the research question. Field notes were written to record the verbal and non-verbal cues from the participants. The interviews were conducted in English and lasted for an average of 30 to 45 min. Consent for using a voice recorder was obtained from the participants to capture all interview discussions. Data collection continued until saturation was reached at a total number of 14 participants, where all the questions had been covered and no newer information could be found from the participants.

#### 2.2.5. Trustworthiness

The concept of credibility, dependability, transferability, and confirmability were applied to ensure trustworthiness in this study. During data collection, experienced qualitative researchers were involved and were responsible for checking the source of data, until data saturation was reached. Research documents, such as the transcripts, field notes, and voice recordings were shared with the independent coder in order to reach consensus on the findings of the study and the interpretation thereof. We arrived at a comprehensive explanation for data in the context of this study, reported herein. Additionally, the researchers did not allow their own opinions to influence the conversations during interviews. The voice recordings and written field notes were shared with the co-coder, who confirmed the data and the general findings.

### 2.3. Ethical Considerations

Ethical clearance was obtained from the Turfloop Research Ethics Committee (TREC/36/2016: PG), as part of a larger study. Permission to collect data at the hospital and clinic was requested from the Limpopo Provincial Department of Health Ethics Research Committee and the Limpopo Capricorn district office. Approval was further requested from the chief executive officer of the hospital and the operational manager of the clinic. The aims and objectives of the study were clearly explained to the participants. Informed consent, with no coercion, was obtained from the participants involved in the study, before data collection could commence. Permission to use a voice recorder was sought before the commencement of data collection. Different codes were allocated to the participants to ensure anonymity, and participants’ information was kept confidential in a safe place.

### 2.4. Data Analysis

Tesch’s eight steps were used for data analysis, as outlined in Creswell [29]. All voice-recordings were transcribed verbatim by the principal researcher. The researcher read through transcripts to get an overall sense of the information obtained. The transcripts were analysed and coded to determine themes and sub-themes that emerged from the data. The independent coder analysed the data separately, and all were in agreement of the themes that represented participants’ responses in order to improve the consistency of the findings.

## 3. Results

Nurses at the hospital were coded as NH (Nurse Hospital), while those at the clinic were coded as NC (Nurse Clinic).

### 3.1. Participants’ Characteristics at Clinic and Hospital, Respectively

A total number of fourteen nurses participated in the study. In the category of age, six participants were aged between 25 and 40 years, three were aged between 41 and 50 years, while five participants were more than 50 years. Among the participants, nine were married, while five were single. Table 1 below reflects the themes and sub-themes that emerged during data analysis.

Three main themes and their sub-themes were derived during Tesch open coding data analysis. The themes are supported by the direct quotations pertaining to nurses’ views regarding breastmilk banking.

### 3.2. Theme 1: Breastmilks Bank and their Importance

Most of the nurses in this study appreciated the launching of the breastmilk bank in the area as a good thing that would help babies who could not breastfeed directly from their mothers. It was highlighted that babies whose mothers were unable to breastfeed would benefit from the donated milk. The following comments were made by the nurses:

*“My view about breastmilk bank is, it is a good idea, because sometimes a mother can deliver a baby and pass on after delivery, and that baby will struggle to have milk. So if the bank is available and there are people who are donating to the bank, then it means those kind of babies will benefit from the bank; then they will grow healthy because we know that breastmilk is good for babies.”*

(NC08).

*“Donated milk from this bank assisted most of the premature. Before the bank was established we used formula, which is not good for them, so donated milk the same as mother’s milk, it is better than formula.”*

(NH05).

*“I would say this bank helped a lot of babies, because there are mothers who are struggling to produce breastmilk—those who are sick and unable to breastfeed their babies. The babies benefit a lot because they receive milk from the bank and they gain weight.”*

(NH03).

*“The human milk bank is a good initiative for the community because it will help by providing breastmilk to children, hence ensuring good growth.”*

(NH01).

Surprisingly, there was one nurse who was interviewed at the clinic who did not know anything about breastmilk banks and specified that she had never heard anything about the new breastmilk bank; the first time she heard about it was during the interview:

*“No, I have never heard anything about breastmilk banks, you are the first person to tell me about it.”* *“* *Let me first understand, this bank is very new? Extremely new and very sensitive, in a sense that we do not know about it, do we have it here? From which kind of mothers will milk be collected, especially now with HIV/AIDS issue? Issues around screening, transportation, and collections might all be a challenge, I think.”*

(NC04).

Nurses at the hospitals where breastmilk bank is available were further asked if they use donated breastmilk in the neonatal ward. All of the participants who were interviewed indicated that donated breastmilk is used when mothers are unable to breastfeed, and it was alluded to that there is a criterion being used for the prescription of donated breast milk:

*“Definitely yes, we use donated milk because some of the mothers have problems with insufficient supply of milk, especially mothers who delivered by caesarean section, and also for babies whose mothers are sick. It is prescribed by doctors.”*

(NH01).

*“We use milk for babies who are unable to receive their mother’s milk. It helps a lot.”*

(NH03).

### 3.3. Theme 2: Donation and Receiving Donated Breastmilk

The issue of donation of breastmilk to the bank revealed different views among the nurses. When asked if they could donate their milk to the bank, most showed positive feelings towards donating:

*“Of course, yes, I would definitely donate my milk to the bank, taking into consideration the benefits of breastmilk to babies who do not have mothers.”*

(NC01).

*“I will definitely donate to the bank, because those babies are in need. Sometimes premature babies suffer a lot, and formula is not good for them because it takes time to digest. Donated breastmilk helps a lot.”*

(NH03).

On the other hand, there was one nurse who seemed not to be interested in donating to the bank due to personal reasons:

*“I don’t think I will donate (silence), I would donate only when there is a serious disaster knowing that my breastmilk will relieve the situation, I don’t think I am fine with breastmilk donation; that is my personal feeling.”*

(NC04).

Nurses were asked if they would receive donated milk from the bank for their children or grandchildren. The discussions regarding receiving donated milk yielded different views from nurses in both institutions. Deliberations highlighted that some of the participants were freely in favour of receiving or using the donated breastmilk from the bank, while others were not:

*“I will definitely receive milk from the bank because I know that milk is tested and safe. They don’t just express and feed other babies straight. They test it like they do with blood. There are machines that they use in the bank. I will accept that milk because I know my baby will benefit from the milk.”*

(NH01).

*“I will accept donated milk to be fed to my baby, because I understand that there is research that was done about donated milk and mothers are tested before they donate. There is no way that they can feed babies milk that is infected, in order to spread infections. I will not have a problem.”*

(NH04).

However, one of the nurses indicated that receiving donated breastmilk could be a traumatic experience as she was once transfused blood and it was stressful afterwards. She always thought about the person who donated blood, having a lot of queries about the donor:

*“I was once transfused blood, and I was so traumatised. I know it helped me a lot but knowing it is someone’s blood, except HIV, I was asking myself, who donated this blood, what did he/she do, is she/he a Christian, will his/her genes be transferred to me? I thought whatever that the donor did will also affect me.” “So I believe with donated breastmilk it will be the same. The donor’s genes could be found in donated milk. I am a health worker I know the process and procedure of screening and cleaning, but as for my baby, no, I will not allow her to feed donated breastmilk.”*

(NH02).

Meanwhile, nurses who were interviewed at the clinic said the following:

*“No, I will not give my child donated breastmilk, but I can donate to the bank; I am not comfortable to give my child breastmilk from another mother. What if the mother who donated milk is sick, not necessarily HIV but any other diseases, then should I give my child that kind of milk? No. Is the milk properly screened?”*

(NC05).

*“If it was my mother I wouldn’t mind her to breastfeed my child. Yes, I know that I will be comfortable with my mother’s breastmilk, but just a stranger, no. It is like you are giving your child to a stranger or somebody else to breastfeed your child. No.” “Honestly speaking, I am a healthcare worker, but don’t trust giving my child someone’s milk. Diseases being my greatest fear, HIV, cancers and so on, I say no. I will not accept my child to be given the donor milk, but I can donate; that is not a problem.”*

(NC06).

During the interviews, culture was also mentioned as one of the factors for not receiving donated milk from the bank:

*“As people, we are not the same; we have different cultural beliefs. In our culture, if I develop ‘sesipidi’ while breastfeeding, then I must stop breastfeeding because ‘sesipidi’ will also be transferred in the breastmilk and the baby will be sick. What if the mother who donated breastmilk has those kind of things; it might affect the use of donated milk, I don’t know.”*

(NC03). “*Sesipidi*” refers to an abscess with pus that can develop anywhere on the body.

*“According to culture, breastmilk belongs to my child only; we cannot share”*

(NC06).

### 3.4. Theme 3: Strategies to Increase Awareness

The discussions with the participants, particularly at the clinic, revealed that nurses were not aware that a breastmilk bank existed in the hospital. This might have a huge impact on the effectiveness of the existing bank. If the nurses are not aware of the existence of the breastmilk, they will not encourage women to donate to the bank, which might lead to rejection of donated breastmilk by the women in the area due to lack of knowledge.

*“* *Mothers might opt for formula feeding rather than using donated breastmilk; the reason for my answer is that I believe this breastmilk bank is a new thing to us so what more about mothers. Therefore it should be thoroughly researched and proven to work. Without training, it will not be effective.”*

(NC04).

After explaining to the nurses that the breastmilk bank exists, most of them appreciated the importance of the breastmilk bank, and they recommended increasing the awareness of the whole community of the Limpopo province.

*“Because it is still a new thing, they need to sensitise us and other health professionals about it. It must be rolled out to the whole community of Mankweng and even the whole province, so that when the mothers visit the clinics or hospitals, they all know that they can donate at the hospital.”*

(NC05).

*“Maybe if we can be educated at the clinic that there is a bank at the hospital, we will then talk about it to mothers during antenatal clinics and explain to them what donated breastmilk is. When they go to hospital and then nurses emphasise it, I think that will be well publicised.”*

(NC04).

A nurse working in the neonatal ward said the following:

*“I would say the government did a very important thing. I realised that most mothers hear about it when they come to the hospital. People in the community must be told about breastmilk banks so that everybody knows about it. If someone is in Vhembe district, he/she must know that Mankweng hospital has a breastmilk bank.”*

(NH01).

Strategies that were suggested by nurses to increase awareness of the existing breastmilk bank included the use of media and health education at primary health care facilities. It was indicated that education about the breastmilk bank should be highlighted during antenatal clinic visits and by using community radio stations.

*“T* *hey need to go out and tell people in the community, maybe by using community radio stations, about breastmilk donation. People must know the importance of the breastmilk bank and its functions.”*

(NH01).

*“Information must reach people, especially during antenatal visits, and media such as community radio stations to ensure that information is provided to large target population who will donate in the near future.”*

(NH04).

The findings revealed that the participants were of the view that provision of information related to the breastmilk bank to the community at large would help to increase the number of donors and recipients of donated breastmilk.

*“I wish people can have information about it and understand how it works, so that they can use the bank if a need arises [laughing].”*

(NC08).

*“Many people lack knowledge and understanding. It doesn’t mean they don’t want to participate. They need information so that they can donate to the bank; they don’t understand the purpose of the breastmilk bank, why is it important.”*

(NC02).

*“The government must try to make sure that every mother in the hospital is well informed by having individual sessions with them explaining the whole process. Then, each and every day, every mother will be encouraged and use a cup to donate the breastmilk.”*

(NH03).

## 4. Discussion

The establishment of the bank was viewed by nurses in this study as a great concept due to the fact that it will assist infants whose mothers are unable to provide breastmilk. Similarly, a study conducted in SA reported that most healthcare professionals are more familiar with the breastmilk bank and that they understand the importance of donated breastmilk [23]. A study in Zimbabwe reported that healthcare professionals accepted the concept of human milk banking [19]. This may be because healthcare professionals understand the importance of breastmilk and breastmilk banks. Surprisingly, there were a few nurses at the clinic who did not know anything about the breastmilk bank. They felt that it was a sensitive issue to deliberate due to HIV being transmitted through breastmilk. The findings from this study concur with results from a study conducted in Nairobi, Kenya, where some healthcare workers at the community level predicted challenges in the establishment of human milk banks due to their safety, cultural reasons, and personal concerns [3]. A study in India reported that almost all of the nurses had insufficient knowledge about breastmilk banking [30]. On the other hand, a study conducted in Punjab, India, reported that most of the nurses who participated in the study had adequate knowledge about the importance of human milk and milk banking [31].

The idea of breastmilk donation and willingness to donate to the existing breastmilk bank was acceptable to most of the participants in this study. In the same way, in South Africa, it was reported that participants were willing to donate breastmilk to the banks [32]. The findings of that study concur with the current findings showing that nurses are willing to donate their breastmilk. The researcher speculated that the participants’ willingness to donate could be motivated by their specific profession as nurses; they understand the importance of breastmilk. In most baby-friendly hospitals in South Africa, healthcare workers undergo about 20 h of a lactation course, which usually emphasises the importance of breastfeeding; therefore, nurses are well informed about the importance of breastfeeding. This might lead to their willingness to donate in order to save lives, showing altruism.

On the other hand, the concept of receiving donated breastmilk was not acceptable to most of the participants in the current study. The major concerns were fear of transmission of disease, notably HIV/AIDS and cancers. Similar findings have been reported in countries such as India and South Africa, where most participants revealed that they would not accept donated human milk to be given to their babies [15,23]. Another study also revealed that there is a high prevalence of HIV infection among the reproductive age in SA; hence, there are perceived dangers associated with breastmilk donation and acceptance. In the current study, there was concern raised by one nurse who related receiving donated milk to blood transfusion, which was a traumatic experience for her. The nurse could not accept donated breastmilk due to a fear of the donor’s genes being transferred into the donated milk. These results are similar to the results from North West (SA), which reported that participants were not in favour of using donated milk due to a fear of transfer of donors’ personal mannerisms to the recipient [21]. In addition, concerns about the screening process were raised by the participants. It was highlighted by some of the nurses that the donor milk should be checked and proven to be safe before being given to their children. Although the nurses were aware of the procedures to be followed before the donation of breastmilk, they seemed not to trust the screening process and believed that “to err is human”. Moreover, some of them might have been influenced by the cultural and religious beliefs that influence the donation and receipt of donated breastmilk. The lack of trust towards the screening processes and the safety procedures at the breastmilk banks might affect the nurses’ acceptance of donated breastmilk to be used for their own children. In a study conducted among mothers and healthcare workers, it was indicated that the delay of acceptability of donated breast milk was due to fear of transmission of infections, particularly regarding the risk of milk containing HIV [23]. Therefore, it is crucial that awareness campaigns be developed to provide reliable evidence about breastmilk banking to healthcare providers and to all members of the community in order to support the established breastmilk banks.

Some nurses believed in cultural reasons for mothers to stop breastfeeding. It is clear that these nurses are concerned about cultural beliefs; therefore, we perceive that even if a nurse experiences some kind of illness related to culture, they would not donate to the bank. Similarly, it was reported that some mothers have the cultural belief that they should stop breastfeeding during sexual intercourse [21]. Moreover, there are fears of long-lasting traditional myths which are still dominant in communities [32]. These beliefs are a hindrance to the donation and acceptability of donor milk. Another study in SA, KZN, supports the findings from the current study, where healthcare workers articulated certain cultural or religious beliefs that could have a huge influence on the acceptability of donated milk [25]. However, in a study conducted in Zimbabwe among healthcare workers, there were no religious issues associated with breastmilk donation [19]. Education is therefore needed to clear the misconceptions regarding breastmilk banking.

On the other hand, nurses in the current study indicated that the established breastmilk bank in Mankweng was not well known by the nurses or the broader community. This could be due to that fact that this study was conducted only a year after the breastmilk bank was established. It is therefore recommended that future studies be conducted to assess if the results would be different from the current study. Hence, it is very crucial for the bank to be well publicised, as suggested by the nurses in the current study. In addition, they suggested the use of media such as community radio stations to increase awareness and education about breastmilk banking during antenatal visits [23]. If nurses have positive attitudes, they are likely to encourage and recruit breastfeeding mothers to donate their breastmilk and to contribute positively to the breastmilk bank [30]. Nurses usually have a great influence on a mother’s breastfeeding initiation by increasing their knowledge about breastfeeding [33]. Furthermore, healthcare workers are the most likely people to encourage mothers to use donated milk [19]. It was also reported that in this area, caregivers mostly rely on healthcare workers, who advise them on accurate information that is most beneficial for their infants [34]. Hospital-based education and increased awareness in the community are definitely imperative, and must occur during antenatal visits [23,34]. In order to reach the maximum donor population, awareness of breastmilk donation should be emphasised in society using numerous means such as mass communication, Non-Governmental Organizations (NGOs), and social clubs, which can play a role in motivating donors [35]. This education should be extended to family members as well as the broader community [23]. Given the uncertainty regarding screening procedures for donors, we call for the development of practical strategies and evidence-based information for nurses, which will assist in promoting and supporting breastfeeding and the established breastmilk banks in the Limpopo Province.

## 5. Conclusions

Almost all nurses at the hospital were knowledgeable about breastmilk banking. However, some nurses at the clinic had insufficient knowledge about breastmilk banking regardless of the bank that was launched over a period of 12 months. Most participants were willing to donate to the bank should a need arise. However, there were hesitations towards receiving donated human milk for their own children, due to the safety of donated milk, screening processes, and cultural issues. Education about human milk banking should be imparted to all healthcare professionals. Awareness campaigns on human milk banking and the processes followed at the bank is imperative to ensure everyone’s buy-in and support of the established breastmilk. This will assist nurses to also motivate mothers to donate and to increase acceptability of the use of donated breastmilk. A larger study exploring views from male nurses, medical practitioners, and other health professionals involved in feeding infants and young children towards human milk banking in Limpopo Province is also recommended.

## 6. Limitations of the Study

The study was limited to one hospital and one clinic in the Mankweng area. The hospital was the only tertiary hospital with an established breastmilk bank during the time of data collection. Furthermore, the study was also limited to one feeder clinic, which serves most of the community around the area. The participants were only female nurses in both institutions. Therefore, the results from this study cannot be generalised to all other clinics, hospitals, and nurses in the Limpopo Province. However, the information gathered from this study could form a basis on which knowledge, attitudes, beliefs, and practices of all healthcare workers about breastmilk banking in Limpopo province are determined.

## Figures and Tables

**Table 1:** Themes and sub-themes that emerged from the data.

Themes	Sub-Themes
Breastmilk banks and their importance	Breastmilk bank viewed as a better option than formula Breastmilk viewed as a way to reduce family costs Usage of donated human milk by nurses
2. Donation and receiving donated breastmilk	Opinions towards donation to the bank Views concerning receiving human milk from the bank Acceptability of donated human milk
3. Strategies to increase awareness	Roll out of a breastmilk bank facility to the community members Use of media to increase awareness Provision of information about human milk donation with the aim of achieving acceptance of the idea

## Data Availability

Not applicable.
